# A cluster-randomized controlled trial assessing the effectiveness of a multifaceted versus a discrete implementation strategy on fidelity to an occupational guideline for the prevention of mental health problems at the workplace: a dual perspective from Swedish schools

**DOI:** 10.1186/s43058-025-00821-x

**Published:** 2025-11-21

**Authors:** Andreas Rödlund, Anna Toropova, Rebecca Lengnick-Hall, Byron J. Powell, Liselotte Schäfer Elinder, Christina Björklund, Lydia Kwak

**Affiliations:** 1 Unit of Intervention and Implementation Research for Worker Health, Institute for Environmental Medicine, Karolinska Institutet, Stockholm, 171 77 Sweden; 2 Center for Mental Health Services Research, Brown School, Washington University in St. Louis, St. Louis, MO USA; 3 School of Public Health, Washington University in St. Louis, St. Louis, MO USA; 4 Division of Infectious Diseases, John T. Milliken Department of Medicine, School of Medicine, Washington University in St. Louis, St. Louis, MO USA; 5 Department of Global Public Health, Karolinska Institutet, Stockholm, 171 77 Sweden; 6 Centre for Epidemiology and Community Medicine, Region Stockholm, Stockholm, 104 31 Sweden

**Keywords:** Implementation science, Implementation strategy, Mental health, Fidelity, Guideline, Primary prevention, Occupational health, Work conditions, Psychosocial risk management, Workplace intervention

## Abstract

**Background:**

Although the management of psychosocial risks in the work environment represents an evidence-based approach to the prevention of mental health problems, its implementation is limited, including in schools, and knowledge on how to support better implementation is scarce. This study compares the effectiveness of a multifaceted vs. a discrete implementation strategy on fidelity to an occupational guideline for the prevention of mental health problems. Dual perspectives were used to assess fidelity, an important aspect of the measurement agenda.

**Methods:**

A cluster-randomized controlled trial was conducted among 55 schools in Sweden. A multifaceted strategy (educational meeting, implementation teams, ongoing training, Plan-Do-Study-Act cycles, and facilitation) was compared with a discrete strategy (teams participating in the educational meeting). Fidelity to the guideline’s recommendations from the recipients’ perspective was measured by questionnaire (Baseline *n* = 2276; 12 months *n* = 1891). Fidelity from the implementers’ perspective (*n* = 54) was assessed via a checklist at 12 months. Linear mixed modeling was used. A qualitative approach was applied to analyze the open-ended responses to the checklist.

**Results:**

Absolute changes in recipient fidelity were observed in all three indicators of the guideline’s recommendation 1 (Multifaceted: 13.2 to 19.5%, Discrete: 10.4 to 13.2%). A statistically significant effect was found favoring the multifaceted strategy (*d* = 0.16). The indicator of recommendation 2 also supported the effect of the multifaceted strategy (Multifaceted: 9.2%, Discrete: 5.0%; *d* = 0.16). The largest difference between the strategies was observed for recommendation 3, for six indicators (Multifaceted: 0.7 to 13.9%, Discrete:—3.2 to 0.0%; *d* = 0.19 to 0.41). Convergence was observed between the two perspectives in support of the multifaceted strategy’s favorable effect on guideline fidelity compared to the discrete strategy. The findings complemented each other, with implementers describing the activities that were enacted and recipients quantifying the change in fidelity over time.

**Conclusions:**

The multifaceted strategy was more effective than the discrete strategy in fidelity attainment after 12 months. Assessing fidelity from the implementer and recipient perspectives provided an understanding of the contextual functioning of the strategies, highlighting the variation in fidelity and the importance of examining the need for adaptations of strategies during the implementation process.

**Trial registration:**

The trial was registered the 9th of August 2021 at Clinicaltrials.gov with Trial registration number: NCT05019937.

**Supplementary Information:**

The online version contains supplementary material available at 10.1186/s43058-025-00821-x.

Contributions to the literature

- The study contributes to the evidence on effective implementation strategies for occupational guidelines to prevent mental health problems at the workplace.
- The study addresses the call for more comprehensive assessments of fidelity as an implementation outcome, including both the implementer and recipient perspectives.
- The study is one of the few randomized controlled trials examining fidelity as a dependent variable when testing the effectiveness of implementation strategies, especially in understudied school settings.
- The comprehensive assessment provides an understanding of the contextual functioning of the strategies and variations in fidelity attainment. It also highlights the potential of strategy adaptations during the implementation.

## Background

### Occupational guidelines to prevent mental health problems at the workplace

Managing psychosocial work environment risks that can lead to stress and mental health problems (MHP), such as high work demands, low work control, and lack of support from colleagues, is one way to prevent MHP in the working population [1]. Workplaces with structured psychosocial risk management systematically identify and assess risks, develop and implement risk reduction action plans, and evaluate and review these actions [1, 2]. Many countries regulate the systematic work environment management through national policies and provisions, but implementation is often limited [2, 3]. Occupational guidelines have been developed to support organizations with their risk management [2]. One example is the “*Guideline for the prevention of mental ill-health at the workplace,*” (further, the Guideline) which includes three recommendations for psychosocial risk management: (a) workplaces should have well-established policies and routines for psychosocial risk management, (b) management should have knowledge of the relationship between psychosocial risks and MHP, and (c) workplaces should regularly assess the psychosocial work environment and intervene on identified risks systematically [4]. This guideline is a complement to the Swedish National provisions for the systematic management of the Work Environment (SAM) [5] and the Organizational and Social Work Environment (OSA) [6].

The mere existence of guidelines, including occupational guidelines, does not guarantee their implementation in practice [7–9]. Systematic reviews on occupational guidelines related to the prevention and management of MHP at the workplace have highlighted the lack of implementation of these guidelines [10, 11]. The guidelines rarely provide practical tools for implementation, and those tools that are provided are often time-consuming and resource-intensive and require additional training [10, 11]. To our knowledge, no studies have tested the effectiveness of implementation strategies on fidelity to occupational guidelines to prevent MHP in the workplace. Research in this area is therefore needed [10, 11].

### Multifaceted implementation strategy for guidelines to prevent MHP

Occupational guidelines to prevent MHP can be considered complex as they consist of several interacting components, involve multiple actors who collaboratively decide on the process and content of activities, may require change at different levels, and allow flexibility in how they are implemented [10–13]. Moreover, multiple contextual determinants influence how effectively guidelines are implemented [14, 15]. Determinants that have been shown to hinder the management of psychosocial risks in the work environment, as recommended in these guidelines, are often related to implementer characteristics, such as insufficient knowledge and motivation, as well as to organizational barriers, including lack of support, guidance, and resources [3, 16–19]. Therefore, implementing such guidelines successfully likely requires a combination of different implementation strategies to address these determinants [10, 11]. However, meta-analyses and systematic reviews have repeatedly reported inconsistent findings on the effectiveness of multifaceted implementation strategies relative to discrete strategies [20–22]. The evidence of the superiority of multifaceted strategies over discrete strategies is therefore mixed, warranting further research on the effectiveness of both discrete and multifaceted strategies [23]. Furthermore, most studies on implementation strategies’ effectiveness have been conducted in a healthcare setting, highlighting the need for more research on the effectiveness of implementation strategies in other settings, such as schools.

Schools have unique context-specific determinants that influence implementation and warrant implementation strategies adapted to the school setting [24, 25]. For example, schools operate on an academic calendar with recurring cycles (e.g., grading periods, holidays, and summer breaks), which challenge the timing and continuity of the implementation [26]. Moreover, in this specific Swedish public school context, school principals are legally responsible for managing psychosocial risks but operate in subordinate positions to their local school district, and they perceive limited decision-making power to influence working conditions for teachers and themselves [27, 28]. These contextual determinants underscore the importance of selecting the implementation strategies based on both the needs of the given effort and the specific setting [29]. Additionally, those implementation studies that have been conducted in a school setting have primarily focused on students’ mental health rather than that of school staff [26, 30, 31]. The success of programs targeting student health, however, heavily relies on the mental health of school staff [26, 30, 31]. This study will address these gaps by testing the effectiveness of a multifaceted strategy for implementing guidelines to prevent MHP among school staff in schools.

### Refinement of a multifaceted implementation strategy for guidelines to prevent MHP

In a cluster-randomized trial conducted in 19 schools between 2017 and 2019 [32], we compared the effectiveness of a multifaceted implementation strategy containing an educational meeting, local implementation teams, ongoing training, and an evaluative and iterative strategy operationalized through Plan-Do-Study-Act (PDSA) cycles on guideline fidelity, with the discrete strategy of the educational meeting alone. Although no statistically significant differences in guideline fidelity were observed between the strategies over time, positive trends favored the multifaceted strategy [33]. For instance, adjusted odds ratios (AOR) indicated higher fidelity for the multifaceted strategy compared to the discrete strategy across five of eight measured indicators (AOR = between 1.01 to 1.70) [33]. Furthermore, when analyses were adjusted for schools characterized by organizational stability, one indicator showed a statistically significant advantage for the multifaceted strategy (AOR = 3.20, 95% CI = 1.40–7.27) [33]. Moreover, when the guideline was implemented with fidelity, improvements in the school’s work environment and better health outcomes among staff were more likely to occur [34]. Given these positive effects on work environment and health, knowledge is needed on how to improve the effectiveness of the multifaceted implementation strategy.

The process evaluation conducted parallel to the trial showed a lack of support from the school districts, which may have impeded the implementation of the guideline [33, 35]. To formalize the school districts’ involvement in the implementation process, the research team decided to add facilitation, defined as – *a process of interactive problem-solving and support that occurs in the context of a recognized need for improvement and a supportive interpersonal relationship* [36] – to the multifaceted strategy [35]. A recent meta-review showed that the success of organizational-level interventions, such as those recommended in the occupational guidelines for the management and prevention of MHP, is dependent on manager support and problem assessment further highlighting the importance of including facilitation [37]. Facilitation through the school district (a form of senior management) may, therefore, be a suitable implementation strategy. Indeed, a lack of expertise and support has been identified as a barrier to psychosocial risk management in a large survey study among European Member States [3]. Facilitation by the school district is expected to support schools with problem identification, action/implementation planning, assessing and monitoring implementation, and goal/priority setting [35]. The structure and processes that are provided through facilitation are expected to improve the school’s opportunity to implement the guideline with fidelity [38–40]. Building on our previous trial, we hypothesize that adding facilitation to the multifaceted strategy will improve the strategy’s effectiveness on fidelity attainment [35].

### Measuring fidelity to occupational guidelines to prevent MHP at the workplace

Among the studies that have examined implementation outcomes as dependent variables when testing the effectiveness of implementation strategies, fidelity—*the degree to which an intervention is implemented as prescribed or intended—*is the most studied [41]. In recent years, significant advancements have been made in the development of measures for fidelity and their psychometric properties [42, 43]. However, there remains a lack of consensus on how to accurately assess fidelity, which calls for improvements in the development and use of high-quality fidelity measures [44]. Improvements pertain, among others, to the quality and comprehensiveness of fidelity assessment, and the implementation properties of mixed-method fidelity assessment [44–46]. To ensure the assessment of fidelity as a multidimensional concept, it has been recommended to measure fidelity from both the implementers’ and recipients’ perspectives [44]. Measuring fidelity from the implementers’ perspectives provides information on the implementation of an intervention (the guideline), including the behaviors enacted, while measuring fidelity from the recipients’ perspectives informs on the engagement and understanding of an intervention [44, 46]. Combining these perspectives will provide a better understanding of the implemented changes and a more granular understanding of fidelity [47, 48]. Addressing fidelity in this manner also allows for a better understanding of the process to achieving implementation effectiveness [49].

Systematic reviews highlight the lack of studies that have tested the effectiveness of implementation strategies on fidelity to occupational guidelines for preventing MHP in the workplace [10, 11]. One reason for this is that it is methodologically challenging to evaluate the implementation of organizational interventions [48] that are recommended in occupational guidelines for the prevention of MHP at the workplace. Organizational interventions are complex, and often involve multiple levels of change, and multiple actors whose involvement changes throughout the process [50]. Actors include senior managers, who provide support through resources [50], middle managers, who are the drivers of change [51] and employees who are active participants in the intervention [52]. Due to the multilevel approach of organizational interventions, it is recommended that the implementation process should be assessed from the perspective of both the managers (implementers) and the employees (recipients) [48, 53]. We have found no studies that have examined the effectiveness of implementation strategies on fidelity to organizational intervention from a dual perspective. The present study presents a unique opportunity to enhance our understanding of how to measure fidelity to the complex organizational interventions recommended by these occupational guidelines.

### Study aims and hypotheses

This study is part of a large cluster-randomized controlled trial conducted among 55 schools in Sweden aimed at exploring the implementation mechanisms through which a discrete strategy (educational meeting and local implementation teams) and a multifaceted implementation strategy (the educational meeting, local implementation teams, ongoing training, PDSA cycles and facilitation) operate to affect fidelity to the “*Guideline for the Prevention of Mental Ill-health at the Workplace.*” The first step in exploring implementation mechanisms and answering implementation questions, such as why, what combination, and in what sequence implementation strategies work, is to test the direct effect of implementation strategies on fidelity. Fidelity was selected as the targeted outcome of these strategies as the critical implementation challenge for schools is to work in accordance with the recommendations provided by the guideline [33]. Planning workshops prior to the first trial showed that schools often have the intention to work systematically with their work environment in accordance with the recommendations of the guideline; however, they experience challenges with its practical implementation. Moreover, it is fidelity to the guideline that leads to practical benefits for the schools, such as improvements in the work environment and staff health [34].

The present study will therefore focus on comprehensively reporting the implementation effectiveness of the strategies, including their ability to address each recommendation and its related target activities. This approach enables a granular understanding of how well the strategy addresses each guideline recommendation and helps identify which aspects may require further attention. The first aim of the current study is to compare the effectiveness of the multifaceted vs. discrete implementation strategy on guideline fidelity. The hypothesis is that schools receiving the multifaceted strategy will report larger improvements in guideline fidelity than schools receiving the discrete strategy. In our previous trial, guideline fidelity was assessed by the school staff only, i.e., the recipients’ perspective [33]. To further our understanding of guideline fidelity, this trial will also assess fidelity from the school management, i.e., the implementers’ perspective, including the forms used to carry out the guideline with fidelity. By comparing and integrating these two perspectives, a more comprehensive assessment of fidelity can be achieved. The second aim of the current study is, therefore, to examine and compare guideline fidelity assessed by recipients and implementers. We hypothesize that there will be an agreement (convergence) between the two perspectives, but that they will also complement each other and will expand our understanding of guideline fidelity. Future studies within the trial will explore the hypothesized implementation mechanisms of the tested implementation strategies.

## Methods

### Trial design

This study is a cluster-randomized waitlist-controlled trial with two arms, including before and after measurements. Schools were randomized (ratio 1:1) to receive either a multifaceted or a discrete implementation strategy. A detailed description of the randomization process is described in the study protocol [35]. Schools were randomly assigned to ARM 1 (multifaceted group) and ARM 2 (discrete group). First, schools within the three municipalities that were recruited first were randomized, stratified by municipality and clusters. To avoid contamination, schools that shared the same principal were grouped into a cluster and randomized together to the same study ARM. In other words, while the school was the unit of analysis, schools under the same leadership (i.e., the same principal) were treated as one cluster for randomization purposes to ensure that implementation activities and leadership decisions did not cross trial ARMs. Upper secondary schools were treated as a separate stratum to guarantee an even distribution across the two groups. Secondly, schools belonging to the fourth municipality were randomized, the same process was applied, also here schools that shared the same principal were clustered and randomized as a cluster. These clusters were placed in a distinct stratum to balance the number of staff. No further stratification criteria were used.

The randomization procedure involved assigning group allocations by randomly ordering the clusters within each stratum. A predefined seed was applied to make the process reproducible. An independent statistician, who was unaware of the schools’ identities and not involved in the project, performed the randomization. The principal investigator (LK) had no influence on the group assignments. For practical reasons, such as coordinating the scheduling of the educational meeting and workshop, randomization was conducted prior to collecting baseline data. Once assignments were finalized, the principal investigator communicated the group placements to the municipalities and schools. Due to the study design, it was not possible to blind school principals or implementation team members to their assigned groups. The trial was approved by the Swedish Ethical Review Agency (2021–01828). The trial was registered the 9th of August 2021 at Clinicaltrials.gov with Trial registration number: NCT05019937 (https://clinicaltrials.gov/study/NCT05019937). Unique Protocol ID: 2020–01214.

### Targeted sites and population

**Fig. 1:**
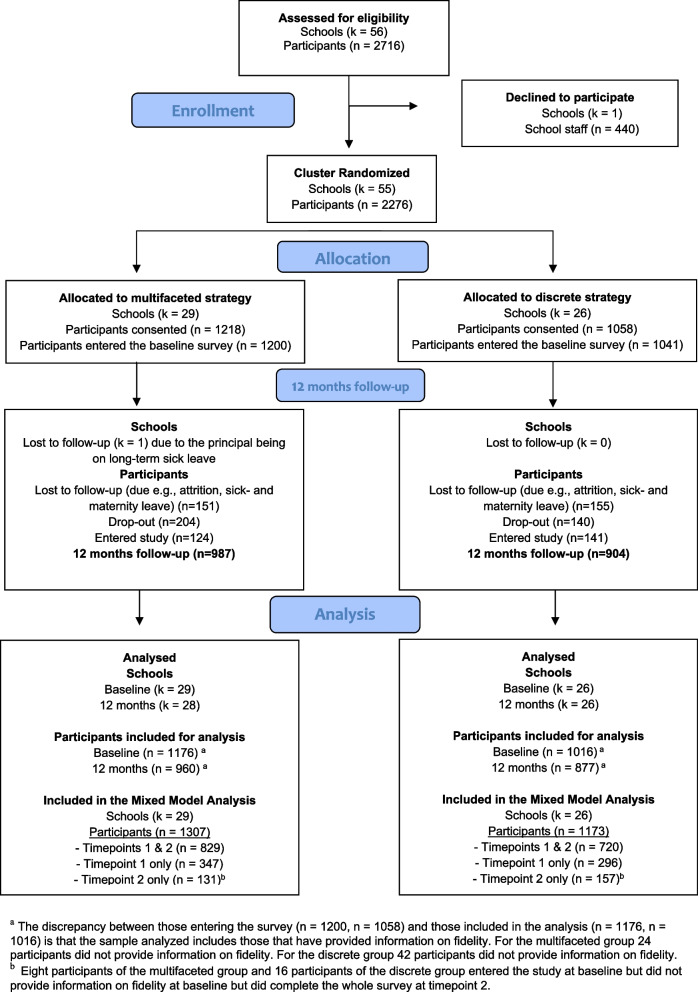
Consort flowchart

The trial was conducted between September 2021- September 2023 in 55 public schools from four municipalities in Sweden. The implementation strategies were delivered between October 2021-June 2022. The participating municipalities were well-represented in relation to sociodemographic and geographical aspects. In Sweden, the municipality ensures that schools comply with steering documents, regulations and laws, including the Swedish Work Environment Act. The school principal coordinates this work at the school level (SFS2010:800). All public schools (*n* = 56) of the municipalities were eligible for participation. One school declined participation due to large organizational changes. Schools varied in school size, ranging from 9 to 180 employees. During the trial, one school belonging to the multifaceted group dropped out due to the principal’s inability to participate. A more detailed description of the recruitment process is available in Kwak et al. [35]. A CONSORT flowchart is presented in Fig. 1.

Within each school, all staff were eligible for participation (e.g., teachers, and support staff). However, staff not employed by the school were excluded (e.g., cleaning staff). Due to high teacher turnover an open-cohort design was applied, meaning that staff not enrolled at baseline could join the trial at 12 months. Exposure to the implementation strategies occurred at team level and not at the staff level, making this an appropriate design.

At baseline, a total of 2716 individuals were invited to participate, of which 2276 consented (participation rate = 83.8%). At 12 months follow-up, 306 participants did not participate due to staff turnover, sickness absence, or maternity leave, and 344 did not respond to the invitation to participate in the follow-up. In total, 2462 individuals were eligible for the 12-month follow-up, of which 1891 participated (participation rate = 76.8%; including 265 new participants), and 1626 of these participated at both time points (response rate = 85.9%).

### The guideline

The *“Guideline for the prevention of mental ill-health at the workplace”* consists of the following three recommendations describing how workplaces can systematically manage psychosocial risk factors within the work environment and prevent MHP: 1) Workplaces have well-established policies and routines for psychosocial risk management; 2) Management have knowledge of the relationship between psychosocial risk factors and MHP; and 3) Workplaces regularly assess the psychosocial work environment and systematically intervene on identified risks. The recommendations involve complex organizational interventions, and often multiple levels of change and multiple actors (e.g., senior managers, middle managers, and employees) [50–52]. The guideline also emphasizes staff involvement in the management of the psychosocial work environment [4]. Each recommendation consists of several target activities presented in Table 1.

**Table 1:** Target activities related to the guideline recommendations

**Recommendation 1: Schools have well-established policies and routines for psychosocial risk management**	
Each school has work-environment documents describing the school’s routines for the prevention of MHP through the management of work-environment risks. These documents are well-known among staff (preferably by involving staff in the development/updating of the documents) and enacted by the school.	*Target activities:* – Develop new documents related to psychosocial risk management. – Revise existing documents related to psychosocial risk management. – Disseminate documents related to psychosocial risk management. – Apply documents related to psychosocial risk management.
**Recommendation 2: School principals have knowledge of the relationship between psychosocial risk factors and mental health problems**	
For schools to intervene on risk factors for MHP within the school’s work environment, each school principal has knowledge on which work-environment risks are common within the school context, how these risk factors relate to MHP and how these factors can be intervened on.	*Target activities:* – Formulate the knowledge the principal needs to have regarding the relationship between psychosocial risks and mental health problems. – Identify appropriate courses that can supply the principal with knowledge on the relationship between psychosocial risks and mental health problems. – Apply for courses that can supply the principal with knowledge on the relationship between psychosocial risks and mental health problems.
**Recommendation 3: Schools regularly assess the psychosocial work environment and intervene on identified risks systematically**	
To prevent MHP within schools, regular assessments are conducted of the school’s work environment. Together with the school staff, action plans are developed that in detail describe which activities need to be undertaken to manage the identified risks. The described activities are executed and followed up.	*Target activities:* – Plan the assessment of the psychosocial work environment. – Assess the psychosocial work environment. – Present the results of the assessment of psychosocial work environment to all staff. – Identity priority areas in group discussions based on the results that need to be intervened upon. – Develop an action plan describing activities aimed at the priority areas. – Execute the intended activities – Continuously follow-up the action plan.

### Implementation strategies

**Fig. 2:**
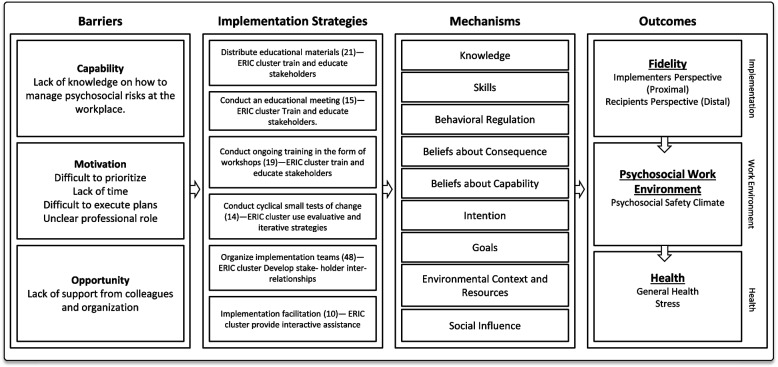
The implementation research logic model for the multifaceted implementation strategy

The multifaceted implementation strategy includes the following components: an educational meeting, a implementation team at each school, ongoing training through workshops, PDSA cycles, and an internal facilitator [35]. The discrete strategy entailed that schools formed an implementation team that participated in the educational meeting the first year and received the remaining strategies after at 12 months [35]. The current study reports from the first year. A detailed description of the development, selection and content of the implementation strategies is provided in the study protocol [35]. The implementation strategies are briefly described in Table 2. The implementation research logic model depicts the theory of change for the multifaceted strategy building on COM-B [54] and the Theoretical Domains Framework [15] (Fig. 2).

**Table 2:** Description of implementation strategies

	**Description of implementation strategies**
Educational meeting	An educational meeting was conducted within each municipality led by an implementation researcher (L.K) and a researcher in occupational health (C.B). Implementation teams and representatives from the municipality (e.g., the director of education and human resource specialists) were invited to attend the educational meeting. The meeting included lectures, discussions, and group exercises. The lectures included information about work-related MHP, the prevention of MHP at the workplace, the Guideline and its recommendations, barriers, and facilitators to implementing and information on how to develop and implement an action plan. During the lectures each implementation team had the opportunity to reflect on their current fidelity to the Guideline and the benefits of working in line with the guideline. The exercises were structured to support the teams with goal formation and developing plans for implementing the Guideline within their school. Implementation teams also received educational material, such as lecture slides and working material, including templates for forming specific, measurable, achievable, realistic, and timely (SMART) goals, as well as material for developing and implementing their action plan.
Implementation Team	Each school was instructed to form an implementation team of 3–5 individuals working at the school (e.g., principal, assistant principal, teacher union representative and occupational health and safety officer). The implementation team’s task was to implement the Guideline within their school. The school principals were responsible for the team’s formation. Instructions, including a template in which principals could specify team members’ names, roles and motivation for inclusion, were provided by the research team to support school principals with the formation of their teams. Implementation teams were encouraged to have regular meetings.
Ongoing training in the form of workshops	Ongoing training was given through five workshops (2.5 h) held over 12 months and led by researchers (L.K and C.B) to support the implementation teams in implementing the guideline. Each workshop was structured in three modules, starting with an update on schools’ activities since the last workshop, during which the teams described and presented their progress in accordance with their PDSA cycle. The second module involved a lecture on the Guideline (one recommendation per workshop) combined with discussions and group exercises. The third module aimed at increasing the implementation team’s skills in implementing the Guideline within their school by using the PDSA methodology in which schools conducted exercises and participated in lectures to make a plan following PDSA and outlining SMART goals. The implementation teams received educational materials such as lecture handouts, PDSA and SMART templates.
PDSA cycles	The implementation teams started their first cycle during the first workshop, when they planned (*plan*) an action related to implementing one of the recommendations of the guideline into their school. The teams were instructed to implement *(do)* their action plan between the workshops. During the next workshop, the implementation team studied *(study)* their progress and revised their plan if needed *(act)*, or if the plan was accomplished, started with a new plan and a new cycle.
Internal Facilitator	Internal facilitators were selected by the municipality. Examples of professions for internal facilitators included the Head of Education, Human resource specialists and school-district principals. The facilitator’s role included supporting the implementation teams of their municipality with problem-solving, prioritizing, and positive reinforcement. Core activities included problem identification, implementation planning, clarifying roles, priority setting, and assessing and monitoring the implementation. Internal facilitators were also instructed to participate in the educational day and workshops.

### Data collection and outcomes

Data were collected in September 2021 (baseline) and September 2022 (follow-up) [35]. Recipient data was collected via an electronic survey to all staff, including teachers and support staff through Research Electronic Data Capture (RedCap) [55]. Implementer data was collected via an electronic checklist to the school principal or assistant principal of each school.

#### Fidelity measured through the recipients’ perspective

The recipient survey included questions on gender, age, educational level, professional title, work experience, work conditions, as well as questions on psychosocial safety climate [56], general health [57], and perceived stress [58]. Moreover, the survey included eleven items to measure guideline fidelity, with ten items on a 5-point Likert scale (1 = strongly disagree, 2 = disagree, 3 = neither agree nor disagree, 4 = agree, and 5 = strongly agree). All items were developed and tested for the purpose of the trials [32, 35]. For this trial, minor modifications to item formulation were made based on cognitive interviews with school staff not participating in the trial (*n* = 5) [35]. Three items measured fidelity to recommendation 1 (α = 0.92), a single item measured fidelity to recommendation 2, and seven items measured fidelity to recommendation 3 (α = 0.91). Items were treated both as summarized as a total score of their recommendations (Range: Recommendation 1 = 3–15, Recommendation 3 = 6–30) and individually, since each item represents a specific target behavior of the guideline and therefore provides granularity to the understanding of fidelity. Given a sufficient number of levels of the Likert scale items as well as an additional use of summed scales, they were treated as interval for the purpose of analysis in this study. Further details about the instrument can be found elsewhere [32, 35].

Furthermore, for recommendation 3, respondents first answered the question: “At our school, an assessment of the psychosocial work environment has been conducted?” with response options (a) Yes, among all employees, (b) Yes, among some employees, (c) Yes, the assessment is ongoing, (d) No, but is planned, and (e) No. If the response was “yes”, six items followed.

#### Fidelity measured through the implementers’ perspective

The checklist measuring fidelity from the implementer perspective was developed for the purpose of the trial. It was pilot tested among principals not participating in the trial. To obtain a common view of psychosocial risk management, school management, together with the health and safety officer and other relevant staff, completed the checklist. The checklist consisted of twelve statements divided into three sections (see Table 1 for target activities).

Each section included statements related to the recommendations' target activities. Section 1 included two statements related to recommendation 1; section 2 included three statements related to recommendation 2; and section 3 included four statements related to recommendation 3. For example, one statement related to recommendation 1: “The school has developed concrete work environment policies that describe routines and practices for how the school prevents MHP among personnel.” Responses to the statement followed the same procedure: (a) the respondent indicated the extent to which they agree/disagree with the statement. If they agree, the respondent was (b) requested to attach documents or material to support their response and (c) requested to describe in an open-ended question the attached document/material, how, when, and by whom the document/material was developed.

### Data analysis

#### Recipient data

Results of the analysis are reported in accordance with the CONSORT guideline for pragmatic and cluster randomized trials (see supplementary material file 1 for CONSORT checklist). To answer aim 1, firstly within-group absolute changes in fidelity for each item of the recommendations were calculated between baseline and 12 months for school staff. Secondly, relative changes in fidelity, which are the adjusted percent change relative to the control mean at post-test were calculated based on observed group means [59]. The changes were classified according to Grimshaw and colleagues [60]: small (< 5%); modest (between 5 and 10%); moderate (between 11 and 20%), or large (> 20%). To assess the degree of clustering in the data at the school level, the intraclass correlation coefficient (ICC) was calculated [61].

Linear mixed models (LMM) were used to analyze changes in fidelity between groups. LMM is a robust approach in modeling treatment effects in an RCT to model repeated measures within a hierarchical data structure, with repeated observations nested within individuals (accounted for by using a person-specific random intercept) and individuals nested within schools (accounted for by a school-specific random intercept) [61]. Separate models were fit for each guideline recommendation item; each item represented a distinct target behavior of the guideline. Additionally, recommendations 1 and 3, which contained several items, were tested using a summarized variable representing the total score of the whole recommendation.

Models were estimated in SPSS (version 28) using restricted maximum likelihood (REML). The change in fidelity between baseline and 12 months was treated as the dependent variable. The first model (M_1_) tested included fixed effects for time and groups, and their interaction (time × group) was used to model the treatment effect. To account for variation across individuals within schools, a random intercept was specified using a “variance components” (VC) covariance structure. Repeated measures across time were modeled with an autoregressive covariance structure (AR1) to address potential autocorrelation. Based on findings from previous studies, we hypothesized that school staff’s work experience at the school could influence the schools fidelity [33]. Therefore, a second model (M_2_) was also tested, which included school staff’s current work experience as a fixed covariate. Results for the treatment effect (time × group) are presented as B coefficients with 95% confidence intervals. In addition, standardized effect sizes (Cohen’s d) were calculated for the treatment effect [62]. The significance level (alpha) was set at 0.05 for two-sided statistical tests.

A power-calculation was conducted for the main research question of the trial and the related mediation analysis, as described in the study protocol [35]. To examine the effect on the implementation outcome presented in this study, we estimated a sample size of around 1500 participants, guided by the results of the previous trial [32, 33].

#### Implementer data

The open-ended responses to the checklist at 12 months were analyzed separately for each recommendation by using a qualitative approach with a manifest interpretation of the data, meaning that the visible, obvious content was categorized with little interpretation [63]. This qualitative approach was applied to explore variation in fidelity by examining how rigorously schools enacted the target activities specified in each recommendation. In the first step, AR and LK, blinded to the school’s allocated implementation strategy, individually read through the open-ended responses. While reading the responses, activities related to the recommendation were highlighted. For recommendation one, activities related to the dissemination of work-environment policies were highlighted, for example: “*during a workplace meeting, the principal talked about the schools’ routines for managing psychosocial risks and showed where the personnel could find the school documents digitally*.” In the second step, schools were classified according to fidelity levels. Based on differences in the schools’ enactment of the guideline recommendations, we identified different fidelity levels (e.g., high, moderate, and low) to reflect meaningful distinctions in guideline fidelity, where each level aimed to represent a distinct degree of rigor of guideline implementation. Fidelity levels were created until no further levels of fidelity could be identified. Schools with high fidelity based on the quality and rigor of the enacted activities, were selected first. After the identification of high-fidelity schools, responses of the remaining schools were read again, to select schools with moderate fidelity. The procedure stopped when there was no more observed variation in the level of fidelity. In the third step, the degree of agreement in categorization between AR and LK was assessed. In case of disagreement, CB and AT were consulted. In the final step, the school’s ARM was unblinded to enable comparison between ARMs both quantitatively (number of schools in each category) and qualitatively (quality and rigor of activities).

#### Integration of implementers’ and recipients’ data

An integration analysis was conducted comparing the data of the implementers and recipients. This analysis served the function of convergence (confirming the validity of the findings), complementarity (contributing with different but non-contradictory findings), and expansion (allowing for further interpretation of the results) [64, 65]. Implementer and recipient data were matched.

## Results

Table 3 presents the baseline characteristics of the schools and school staff (recipients) in the multifaceted and discrete strategy groups. The proportion of females was around 71% in both groups. The average age of participants was 48.4 years in the multifaceted group and 48.7 years in the discrete group. Most participants had a university education (73.5% in both groups). In the discrete group 63.4% of participants were teachers compared to 61.3% in the multifaceted group. Most participants, approximately 93%, had permanent employment, and the majority worked full-time. Approximately 27.5% of participants reported working overtime at least a couple of days a week. In the discrete group, 33.9% of participants reported very good health compared to 30.3% of participants in the multifaceted group.

**Table 3:** Baseline characteristics of schools and recipients in the multifaceted and discrete strategy groups

Characteristics	**Multifaceted Group**			**Discrete Group**		
	**n**	**%**	**Mean (SD)**	**n**	**%**	**Mean (SD)**
**Schools**	**29**			**26**		
Municipality						
Rural municipality^b^						
Municipality 1	4	13.7		4	15.3	
Municipality 2	6	20.6		5	19.2	
Commuter municipality near a major city^b^						
Municipality 3	14	48.2		11	42.3	
Municipality 4	5	17.2		6	23.0	
School Type						
Primary School (Students aged 6 to 12 years)	19	65.5		18	69.2	
Secondary School (Students aged 13 to 16 years)	7	24.1		6	23.0	
Upper Secondary School (Students aged 16 to 19 years)	3	10.3		2	7.6	
Staff per school			51 (39)			48 (38)
**Staff**	**1218**^**a**^			**1058**^**a**^		
Gender (% Female)	1200	70.7		1041	71.0	
Age	1180		48.4 (10.6)	1032		48.7 (11.0)
Education level	1199			1041		
Basic Education	64	5.3		50	4.8	
Secondary Education	219	18.0		207	19.9	
University Education	895	73.5		785	73.5	
Post-graduate Education	21	1.7		19	1.8	
Professional title	1200			1041		
Teacher	736	61.3		660	63.4	
Other school personnel^c^	464	34.7		381	36.6	
Within the occupation (years)	1191			1034		
Less than 5 years	221	18.6		191	18.5	
5–14 years	364	30.6		325	31.4	
15–24 years	367	30.8		310	30.0	
25–34 years	173	14.5		147	14.2	
35 or more years	66	5.5		61	5.9	
At the school (years)	1193			1035		
Less than 5 years	536	44.9		457	44.2	
5–14 years	434	36.4		377	36.5	
15–24 years	181	15.2		149	14.4	
25–34 years	39	3.3		45	4.4	
35 or more years	3	0.3		6	0.6	
Employment terms	1197			1041		
Permanent	1123	93.8		969	93.1	
Other	74	6.2		72	6.9	
Ordinary working hours	1197			1041		
Full-time (9 h a day)	991	82.8		891	85.6	
Part-time	206	17.2		150	14.4	
Working overtime	1195			1040		
Each day	108	9.0		82	7.9	
Couple of days a week	330	27.6		286	27.5	
Once a week	108	9.0		113	10.9	
Couple of days a month	232	19.4		197	18.9	
More rarely	280	23.4		225	21.6	
Never	137	11.5		137	13.2	
Psychosocial Safety Climate (Mean, Range 0–16)	1149		7.06 (3.77)	998		7.05 (4.04)
General Health	1147			998		
Excellent	101	8.8		83	8.3	
Very good	348	30.3		338	33.9	
Good	456	39.8		362	36.3	
Fairly	215	18.7		183	18.3	
Bad	27	2.4		32	3.2	
Perceived Stress	1148			998		
Not at all	108	9.4		86	8.6	
Some	243	21.2		220	22.0	
To some extent	389	33.9		322	32.3	
Pretty much	278	24.2		240	24.0	
Very much	130	11.3		130	13.0	

^a^*n* = 18 (multifaceted group), *n* = 17 (discrete group) informed consent but not completed the rest of the survey

^b^Municipalities are classified according to the Swedish Association of Local Authorities and Regions municipal grouping. *Rural municipality* = Fewer than 15,000 inhabitants in the largest urban area, with low commuting patterns. *Commuter municipality near a major city* = At least 40 percent of the workforce commutes to a larger city

^**c**^School administrators, recreational pedagogues etc.

### Fidelity from the recipients’ perspective

Absolute within-group changes in fidelity to the recommendations from baseline to 12-month follow-up are shown in Table 4. Schools in the multifaceted group increased fidelity to all items (absolute change in recommendation 1: range 13.2–19.5%; recommendation 2: 9.2%; recommendation 3: range 0.7–13.9%). Schools in the discrete group increased fidelity to recommendation 1 (10.4–13.2%) and recommendation 2 (5.0%), however for recommendation 3, fidelity only increased for one item, while it decreased or sustained for the rest of the items (−3.2—6.0%). The relative changes for the items of recommendation 1 ranged from 2.74 to 6.02%; for recommendation 2 the relative change was 4.16% and for the items of recommendation 3 the relative changes ranged 4.17–13.62%. All observed changes indicated improvements in the multifaceted group relative to the changes observed in the discrete group. For Recommendation 1, the ICC was 0.11 (SE = 0.02), while the ICCs for individual items were 0.08 (SE = 0.01) for item 1A, 0.07 (SE = 0.01) for item 1B, and 0.13 (SE = 0.02) for item 1C. The ICC for Recommendation 2 was 0.11 (SE = 0.02), and for Recommendation 3, the ICC was 0.12 (SE = 0.03). Within Recommendation 3, individual item ICCs were as follows: 0.07 (SE = 0.02) for item 3B, 0.06 (SE = 0.01) for item 3C, 0.11 (SE = 0.02) for item 3D, 0.13 (SE = 0.03) for item 3E, 0.12 (SE = 0.02) for item 3F, and 0.13 (SE = 0.03) for item 3I.

**Table 4:** School personnel fidelity to the guideline recommendations at baseline and 12 months

	**Multifaceted Group**			**Discrete Group**			
	**Baseline (k = 29 *n* = 1176) Mean (SD)**	**Follow-up (k = 28** ***n* = 960)** **Mean** **(SD)**	**Absolute change within group (%)**	**Baseline** **(k = 26** ***n* = 1016)** **Mean** **(SD)**	**Follow-up** **(k = 26** ***n* = 877)** **Mean** **(SD)**	**Absolute change within group (%)**	**Adjusted percent change relative to the control mean at post test**
Recommendation 1: Schools have well-established policies and routines for psychosocial risk management	8.61 (3.1)	9.97 (3.0)	15.7	8.41 (3.3)	9.39 (3.0)	11.6	4.04%
1A. I recognize the content our schools work environment documents	2.86 (1.1)	3.29 (1.0)	15.0	2.81 (1.1)	3.13 (1.0)	11.3	3.51%
1B. I act in accordance with the school’s work environment documents	3.02 (1.1)	3.42 (1.0)	13.2	2.97 (1.1)	3.28 (1.0)	10.4	2.74%
1C. The school has carried out efforts for staff to recognize the content of the work environment documents	2.72 (1.1)	3.25 (1.1)	19.5	2.64 (1.1)	2.99 (1.1)	13.2	6.02%
Recommendation 2: Principals have knowledge of the relationship between psychosocial risk factors and mental health problems							
2A. School/work management has sufficient knowledge and competence in the area of psychosocial risks	3.02 (1.1)	3.30 (1.0)	9.2	2.97 (1.1)	3.12 (1.1)	5.0	4.16%
Recommendation 3: Schools regularly assess the psychosocial work environment and intervene on identified risks systematically	17.94 (5.5)	19.52 (5.7)	8.8	18.21 (5.9)	17.92 (5.8)	−1.5	10.43%
3A. An assessment has been carried out at the school	46.8%	55.7%	8.9	44.2%	50.2%	6.0	5.77%
3B. The result of the assessment was communicated to the staff	4.01 (1.0)	4.04 (1.0)	0.7	3.97 (1.1)	3.84 (1.1)	−3.2	4.17%
3C. Affected staff received an opportunity to participate in a discussion about the results of the assessment	3.47 (1.2)	3.68 (1.1)	6.0	3.51 (1.2)	3.40 (1.2)	−3.1	9.41%
3D. A joint action plan with actions was created with affected staff based on the results of the assessment	2.73 (1.1)	3.11 (1.2)	13.9	2.79 (1.2)	2.79 (1.1)	0.0	13.62%
3E. The action plan was used to follow up that planned actions were carried out	2.64 (1.1)	2.99 (1.1)	13.2	2.70 (1.2)	2.69 (1.1)	−0.3	13.38%
3F. The planned actions were carried out	2.60 (1.0)	2.91 (1.1)	11.9	2.67 (1.1)	2.66 (1.1)	−0.3	12.03%
3I. The actions carried out led to improvements in the psychosocial work environment	2.50 (1.0)	2.79 (1.1)	11.6	2.57 (1.1)	2.55 (1.0)	−0.7	12.16%

Absolute change is classified as follows: small (< 5%); modest (between 5 and 10%); moderate (between 11 and 20%), or large (> 20%). Percentages in indicator 3 A represent school staff who responded that an assessment had been carried out among all or some staff (*Multifaceted Group, Baseline* = *551 and 12 months* = *535*, and *Discrete Group, Baseline* = *450 and 12 months* = *441*) and received items 3B to 3I. Range for individual items: 1–5, Recommendation 1: 3–15, and Recommendation 3: 6–30

The comparative effectiveness of the multifaceted versus the discrete implementation strategy on fidelity to the guideline recommendations and related target activities are presented in Table 5. For recommendation 1 overall, a significant intervention effect was observed (*B* = 0.34, 95% CI: 0.05 to 0.63, *p* = 0.02). This indicates that for the multifaceted group, the change in fidelity to Recommendation 1 from baseline to follow-up was significantly greater compared to the discrete group. When assessing individual items of Recommendation 1, a significant intervention effect was found for item 1C (*B* = 0.17, 95% CI: 0.05 to 0.28, *p* = 0.00), indicating that schools receiving the multifaceted strategy experienced a significantly greater change in fidelity compared to the discrete group over time. For items 1A and 1B, there was no significant intervention effect (Item 1A: *B* = 0.09, 95% CI: −0.00 to 0.20, *p* = .06; item 1B: *B* = 0.07, 95% CI: −0.03 to 0.18, *p* = 0.18). When school staffs’ work experience at the school was introduced as a fixed covariate in the second model (M_2_), the intervention effect for Recommendation 1 was non-significant (*B* = 0.29, 95% CI: −0.00 to 0.59, *p* = 0.05). While the effects for item 1C remained significant (*B* = 0.15, 95% CI: 0.04 to 0.27, *p* = 0.00) and items 1A and 1B remained non-significant.

**Table 5:** The comparative effect of the multifaceted vs. the discrete implementation strategy on fidelity to the guideline with work experience as a covariate

	**n (k)**	**M**_**1**_**: Estimate (S.E)**	**CI 95%**	***d***	**p = **	**M**_**2**_**: Estimate (S.E)**		**CI 95%**	***d***	**p = **
Recommendation 1: Schools have well-established policies and routines for psychosocial risk management	2473 (55)	0.34 (0.14)	0.05–0.63	0.16	0.02	0.29 (0.15)		−0.00 – 0.59	0.14	0.05
1A. I recognize the content our schools work environment documents	2480 (55)	0.09 (0.05)	−0.00 – 0.20	0.13	0.06	0.07 (0.05)		−0.03 – 0.18	0.10	0.17
1B. I act in accordance with the schools’ work environment documents	2474 (55)	0.07 (0.05)	−0.03 – 0.18	0.09	0.18	0.05 (0.05)		−0.05 – 0.17	0.07	0.30
1C. The school has carried out efforts for staff to recognize the content of the work environment documents	2473 (55)	0.17 (0.05)	0.05–0.28	0.21	0.00	0.15 (0.05)		0.04–0.27	0.14	0.00
Recommendation 2: Principals have knowledge of the relationship between psychosocial risk factors and mental health problems										
2A. School/work management has sufficient knowledge and competence in the area of psychosocial risks	2473 (55)	0.12 (0.05)	0.02–0.23	0.16	0.01	0.11 (0.05)		0.00–0.22	0.14	0.04
Recommendation 3: Schools regularly assess the psychosocial work environment and intervene on identified risks systematically	1470 (55)	1.60 (0.39)	0.82–2.38	0.43	<.001	1.59 (0.40)		0.80–2.38	0.44	<.001
3B. The result of the assessment was communicated to the staff	1479 (55)	0.15 (0.08)	−0.01 – 0.32	0.19	0.07	0.17 (0.08)		0.00–0.34	0.21	0.04
3C. Affected staff received an opportunity to participate in a discussion about the results of the assessment	1478 (55)	0.27 (0.09)	0.09–0.46	0.28	0.00	0.29 (0.09)		0.10–0.48	0.31	0.00
3D. A joint action plan with actions was created with affected staff based on the results of the assessment	1473 (55)	0.32 (0.08)	0.15–0.49	0.40	<.001	0.32 (0.08)		0.15–0.49	0.41	<.001
3E. The action plan was used to follow up so that planned actions were carried out	1472 (55)	0.31 (0.08)	0.15–0.46	0.41	<.001	0.29 (0.08)		0.13–0.45	0.39	<.001
3F. The planned actions were carried out	1470 (55)	0.27 (0.07)	0.12–0.42	0.37	<.001	0.27 (0.07)		0.11–0.42	0.37	<.001
3I. The actions carried out led to improvements in the psychosocial work environment	1470 (55)	0.25 (0.07)	0.10–0.40	0.37	<.001	0.25 (0.07)		0.10–0.40	0.37	<.001

n = the number of observations included in the model (k = number of schools included in the model). B = reports time x group. M_1_ = include time, group, time x group as fixed factors, and school x ID as a random intercept. M_2_ = include time, group, time x group, and current work experience at school as fixed factors, and school x ID as a random intercept. *d* = Standardized effect size Cohen’s *d*. A positive estimation indicates larger improvement in multifaceted schools

Recommendation 2 was assessed using a single item. There was a significant intervention effect (*B* = 0.12, 95% CI: 0.02 to 0.23, *p* = 0.01), indicating greater change in fidelity to Recommendation 2 in the multifaceted group from baseline to follow-up. In Model 2 (M_2_), the effect remained significant (*B* = 0.11, 95% CI: 0.00 to 0.22, *p* = 0.04).

For recommendation 3, the intervention effect was significant, with an estimate of 1.60 (95% CI: 0.82 to 2.38, (*p* = < .001). This indicates that for the multifaceted group, the change in fidelity to Recommendation 3 from baseline to follow-up was significantly greater compared to the discrete group. Within Recommendation 3, schools in the multifaceted group showed higher fidelity for item 3B, although this was not statistically significant (*B* = 0.15, 95% CI: −0.01 to 0.32, *p* = 0.07). Significant intervention effects were observed for items 3C through 3I, indicating greater changes in fidelity from baseline to follow-up in the multifaceted group. Specifically, items 3D (*B* = 0.32, 95% CI: 0.15 to 0.49, *p* = < .001) and 3E (*B* = 0.31, 95% CI: 0.15 to 0.46, *p* < .001) showed the largest effect sizes. In Model 2 (M_2_), all items for Recommendation 3 were significant, supporting the hypothesis that the multifaceted strategy was more effective than the discrete strategy.

### Fidelity from the implementer perspective

The fidelity levels as assessed with the checklist, including the number of schools within the multifaceted and the discrete group within each fidelity level, are depicted in Table 6.

For recommendation 1, four fidelity levels were identified. Seven schools (multifaceted group *n* = 4; discrete group *n* = 3) were categorized as having high fidelity. These schools enacted all target activities continuously throughout the year with staff involvement. For example, the schools updated existing documents, described how documents were disseminated, and which activities were enacted to ensure compliance with the document. Eight schools (multifaceted group *n* = 7; discrete group *n* = 1) were categorized as having moderate fidelity. These schools enacted all target activities, however lacked a description of how staff were involved in these activities. Thirteen schools (multifaceted group *n* = 7; discrete group *n* = 6) were categorized as having modest fidelity. These schools lacked a clear description of concrete activities. Seventeen schools (multifaceted group *n* = 8; discrete group *n* = 9) were categorized as having low fidelity, these schools only enacted one activity. The remaining schools did not achieve fidelity to this recommendation.

For recommendation 2, three fidelity levels were identified. Seven schools (multifaceted group *n* = 5; discrete group *n* = 2) were categorized as having high fidelity. These schools enacted all target activities, including formulating knowledge requirements for school principals, suggesting and participating in relevant courses. Twelve schools (multifaceted group *n* = 4; discrete group *n* = 8) were categorized as having moderate fidelity. The management of these schools had not participated in any relevant courses. Twelve schools (multifaceted group *n* = 8; discrete group *n* = 4) were categorized as having low fidelity and only enacted one target activity. The remaining schools did not achieve fidelity to this recommendation.

For recommendation 3, four fidelity levels were identified. Twelve schools (multifaceted group *n* = 10; discrete group *n* = 2) were categorized as having high fidelity. These schools enacted all target activities; they involved staff when presenting the results of their work environment surveys and in the development of action plans. Moreover, they described concrete actions in their action plan, and how they worked with and/or followed up on their action plan. Six schools (multifaceted group *n* = 2; discrete group *n* = 4) were categorized as having moderate fidelity. These schools conducted nearly all target activities, but concrete actions were not included in their action plan. Thirteen schools (multifaceted group *n* = 8; discrete group *n* = 5) were identified as having modest fidelity. These schools described an action plan; however, they lacked a description of staff involvement, concrete actions, and action plan follow-up. Nine schools (multifaceted group *n* = 4; discrete group *n* = 5) were categorized as having low fidelity. These schools only stated that they assessed their work environment and presented the results to their staff. The remaining schools did not achieve fidelity to this recommendation.

### Integration of implementers and recipient results

The examples below illustrate how the recipient and implementer perspectives complement each other. The recipient perspective showed an improvement in item 1C (absolute change multifaceted group = 19.5%, discrete group = 13.2%), meaning that the schools had carried out activities to increase awareness of the work environment documents (Table 4). From the implementers’ perspective, 14% (*n* = 4) of schools in the multifaceted strategy group and 11% (*n* = 3) in the discrete group achieved high fidelity (Table 6). In total, 92% (*n* = 26) of schools in the multifaceted group reached fidelity at any level (high, moderate, modest, or low), while 73% (*n* = 19) in the discrete group reached fidelity at any level (Table 6). In line with this, the implementers’ perspective showed that, independent of what strategy they received, high-fidelity schools disseminated and discussed these documents continuously through staff meetings. For example, in the following citation: *“Ongoing during three staff meetings during the year. First, assessing how many know the content—next, revising and feedback. Lastly, presenting the new* [document] *with a survey on knowledge about routines”* (Table 6). This illustrates how high fidelity schools actively involve staff in establishing their documents, moving beyond merely having a document, which is an essential target activity for working in accordance with recommendation 1. Both the recipient and implementer perspectives showed greater fidelity to this recommendation in the multifaceted strategy group (Tables 5 and 6).

The recipient perspective showed an improvement in item 2A (absolute change multifaceted group = 9.2%, discrete group = 5.0%), meaning that school management has sufficient knowledge and competence in psychosocial work environment risks (Table 4). From the implementers’ perspective, 17% (*n* = 5) of schools in the multifaceted group and 7% (*n* = 2) of the discrete group achieved high fidelity to recommendation 2 (Table 6). In total, 60% (*n* = 17) in the multifaceted group and 53% (*n* = 14) in the discrete group reached fidelity at any level (high, moderate, low) (Table 6). The implementers’ perspective further showed that high-fidelity schools specified the risk management knowledge the school management needs to have and recommended relevant courses. The management of these high-fidelity schools also participated in these courses. One of the schools described their work as follows: “*Plans* [knowledge requirements] *have existed for a long time and are updated before each new school year. We regularly and continuously get invitations through human resources to different work environment courses. The health and safety officer and union representative are also invited, often together with us from school management”* (Table 6). This reflects how high-fidelity schools have established structures to secure and maintain risk management competence within the school, ensuring that knowledge requirements are clearly defined, and regularly updated as recommended in the guideline. Results further showed that the multifaceted strategy was more effective in improving fidelity to recommendation 2, as evidenced by both the recipient’s perspective (Table 5) and the implementer’s perspective (Table 6).

**Table 6:** Qualitative findings of implementers demonstrating levels of fidelity with the corresponding descriptions and practical examples

			**Multifaceted group**		**Discrete group**	
	**Description activities**		**Example reflecting level of Fidelity**	**N**	**Example reflecting level of Fidelity**	**N**
**Recommendation 1: Schools have well-established policies and routines for psychosocial risk management**						
**High Fidelity**	These schools describe all target activities, including continuous dissemination activities through staff meetings. Staff are involved in the development of the documents. Schools have described concrete actions to ensure that the school complies with the documents		School 49 has updated existing documents: *They were revised during the year and came into effect in June 2022. We do not know when they were revised before. These are our work environment routines*. Has described how the document is disseminated:* Ongoing during three staff meetings during the year. First, assessing how many know the content—next, revising and feedback. Lastly, presenting the new* [document] *with a survey on knowledge about routines*. Has described activities that ensure the school complies with the document: *Continuously throughout the year, at staff meetings and during meetings with employer and union representatives. Went through* [document] *during staff meeting with all staff and during meetings with employer and union representatives to ensure that we do everything we say we should be doing, staff meeting 9/3 2022*	4	School 40 has updated existing documents: *During the fall term each year, we go through our documents and guidelines and update them. Annual wheel for systematic quality work*. Has described how the document is disseminated: *The most recent was at the staff conference in August and at the staff meeting on the 6th of September 2022.* [We] *go through* [the documents] *with presentation and discussion at staff meeting the 6th of September 2022.* Has described how it is ensured that the school complies with the document: *Happens continuously through meetings at the school-management level, municipality-level, school-district level, and school-level. And through meetings with human resources and performance management dialogues.* [We]* go through policies, annual wheel, routines, and guidelines as well as quality analysis and employee survey*	3
**Moderate fidelity**	These schools work continuously with the dissemination of documents, however, lack a clear description of how school staff are involved. Schools describe how staff are involved in ensuring that the schools comply with documents, however not which activities are applied in the process		School 53 has updated existing documents: *During the summer of 2022. Plan for systematic work environment work.* Has described how the document is disseminated: *Ongoing through different information meetings, staff in-service days, staff meetings, unit meetings. Both orally, with PowerPoint, and in writing.* Has described how it is ensured that the school complies with the document: *Ongoing during the school year. In conjunction with performance management dialogues, which are twice per school year*	7	School 21 has developed new documents and updated existing documents: *Previous schoolyear. For example, various things related to the pandemic, which were highly relevant the previous school year, but also documents related to routines and policies, e.g., students on medication at school (self-care)*. Has described how the document is disseminated: *Continuously orally and written through weekly newsletter, alternatively staff meetings. Continuously orally and written through* [different staff and leadership meetings]*.* Has described how it is ensured that the school complies with the document: *Continuously orally and written through* [different staff and leadership meetings]	1
**Modest fidelity**	These schools describe several target activities. Dissemination activities are described however not as a continuous process, but more as a one-off activity. A lack of description of concrete activities describing how schools ensure that the school complies with the document		School 54 has updated document: [work environment document] *was updated in August 2022. The work environment plan has been updated according to the annual wheel*. Has described how the document is disseminated: *By going through it* [work environment document] *together during our staff in-service day in August*. Has described how it is ensured that the school complies with the document: *Continuously during the year through an annual wheel, there we ensure that what is stated continues*	7	School 45 has updated documents: *A work environment strategy that is updated annually (common* [document] *for school district)*. Has described how the document is disseminated: *Introduction during schoolyear, 22/23, urging all to read* [the document]*, allocated time see above*. Activities specified to ensure that the document is applied: *Continuously through conversations and discussions*	6
**Low fidelity**	These schools describe one target activity mostly related to the dissemination of documents. A description is lacking how schools ensure that the documents are applied in practice		School 22 has not developed/revised documents, well-functioning documents are already in place. Has not specified activities that will ensure compliance with the documents. Has described how the document is disseminated: *Since the autumn term/2021 when we started the education* [educational meeting and workshops]*. During staff meetings we went through where the documents can be found and how to find them digitally*	8	School 33 has not had the possibility to develop/update documents. Has not specified activities that will ensure compliance with the documents. Has described how the document is disseminated: *August 2022, in conjunction with the start of the school year. Went through* [the document] *with all staff, staff conference*	9
**Recommendation 2: School principals have knowledge of the relationship between psychosocial risk factors and mental health problems**						
**High fidelity**	These schools describe all target activities. Formulated knowledge requirements for the school management within their municipality. Offer courses to provide this knowledge. Schools have also described that their school management have participated in such courses		School 53 has formulated knowledge requirements: *Plans have existed for a long time and are updated before each new school year.* Courses are described: *We regularly and continuously get invitations through human resources to different work environment courses. The health and safety officer and union representative are also invited, often together with us from school management*	5	School 43 started with formulating knowledge requirements: *When we started working with you at* [the university]. Courses are described: *Basic courses and continuous additional courses are available within the municipality for both union representatives and school management*	2
**Moderate fidelity**	These schools describe two target activities. Schools have or have started formulating knowledge requirements for school management. Schools describe courses offered to provide this knowledge; however, not whether school management has participated in these courses		School 4 has formulated knowledge requirements: *School year 21/22*. Courses are described: *Work environment courses for school management and health and safety officer*	4	School 40 has formulated knowledge requirements: *Is formulated in the municipality’s employee policy”*. Courses are described: *Centrally organized SAM* [systematic work environment] *courses”*	8
**Low fidelity**	These schools describe one target activity related to the courses that are offered to provide this knowledge		School 3 has not formulated knowledge requirements. Courses are described: *the employer provides courses through Suntarbetsliv* [a non-profit organization run by union- and employer representatives of the Swedish Municipality and Regions]	8	School 8 has not formulated knowledge requirements. Courses are described: *Work environment courses for health and safety officers and school management*	4
**Recommendation 3: Schools regularly assess the psychosocial work environment and intervene on identified risks systematically**						
**High fidelity**	These schools describe all target activities. They have involved their staff when presenting the results of their work-environment surveys, and in the formation of action plans. Schools have described concrete actions in the action plan, how they work with the action plan and/or follow up the actions described	School 5 has conducted a work environment assessment among all staff: *Autumn term 2021 and spring term 2022, as well as autumn term 2022. The human resource-unit send out an employee survey and Pulse survey to all employees in November 2021 as well as May 2022.* Results were presented to all staff: [By] *school management and collaborative teams.* Action plan was developed together with relevant staff: *Action Plan Employee Survey [attached document]*. The school has carried out the action plan: *We still work with communication and that we are each other’s work environment. Some parts were improved in the Pulse survey.* The school follows up on the action plan: *In June 2022, as well as in November 2022, when the new employee survey is presented*		10	School 29 has conducted a work environment assessment among all staff: *During 2022. Each work team.* Results were presented to all staff: At staff meeting during 2022. [By] *assistant principal during staff meeting*. Action plan was developed together with relevant staff: *Short-term goals were created with respective work team*. The school has carried out the action plan: *The work teams have themselves executed their changes for 2022.* The school follows up on the action plan: *We follow-up our short-term goals during staff meeting, the staff have themselves been involved in deciding how to follow-up*	2
**Moderate fidelity**	These schools describe all target activities. They have involved their staff when presenting the results of their work-environment surveys, and in the formation of action plans. Schools have described actions in the action plan, how they work with their action plan and follow up the plan. However, a description of concrete actions is lacking	School 50 has conducted a work environment assessment among all staff: *Employee surveys during autumn and spring.* [External company] *on behalf of the municipality*. Results are presented to all staff: *Next month when the results* [of the survey] *have come. Principal and assistant principal*. Action plan was developed together with relevant staff: *Developed in November and staff were included the same month during a staff-meeting*. The school has carried out the action plan: *From November to June”*. The school follows up on the action plan: *Staff meeting, performance management dialogues and other meetings*		2	School 40 has conducted a work environment assessment among all staff: *September, February, staff meeting every 6th week. Municipality conducts the employee survey; school has staff meeting.* Results are presented to all staff: *May, August*. *School management orally and presentation during staff meeting.* Action plan was developed together with relevant staff: *After we went through it* [the results] *during staff meeting with all personnel in May, action plans were developed in the respective work teams and before the quality analysis in June.* The school has carried out parts of the action plan: *Started in May and is ongoing.* The school follows up on the action plan: *Staff meeting, operative school management meetings and school management meetings at each unit as well as at management meeting within the school-district*	4
**Modest fidelity**	These schools describe several target activities. An action plan has been developed. However, these schools did not involve their staff, nor have they described concrete actions or how to follow up the action plan	School 52 has conducted a work environment assessment among all staff: *At the employee survey in September/October 2021 and with a follow-up employee survey with selected questions in February 2022. Performance management dialogues in September 2021 and follow-up dialogues in February 2022*. Results are presented to all staff: *The employee survey results for the respective work team were presented at the work team meeting in January/February. The employee survey results for the whole school were presented at staff in-service days in January. In work teams, the school management and work team leader. For the school, the principal.* The school has started with the development of an action plan without involvement of staff: *22-Jan*. The school has carried out the action plan: *staff meetings* [the action in the action-plan] *with all staff have been implemented starting September 2022*. The school does not follow up on the action plan		8	School 26 has conducted a work environment assessment among all staff: *In conjunction with performance management dialogues and a major employee survey with HME* [municipality-level survey] *June-22 among all employees*. Results are presented to all staff: *presented at the start of the school year in August 2022 by the principal*. The school has started with the development of an action plan without involvement of staff: *The conference system* [technical equipment] *has changed a bit, no specific action plan*. The school has carried out the action plan: *August/September 2022*. The school follows up on the action plan: *during staff-meeting with all staff, during work team meeting, in the school-management group*	5
**Low fidelity**	These schools describe that they have assessed the psychosocial work environment and presented the results	School 36 has planned a work environment assessment: *For spring 2023. Central from the administration* [Municipality] and conducted a work environment assessment: *spring 2021 (24-month interval).* Results are presented to all staff: [By] *school management orally and in text.* Action plan was developed together with relevant staff: *mostly on an individual level.* The school has carried out the action plan: *during the year.* The school follows up on the action plan: *Individual plans are followed up by the first-line manager. In some cases, in support of human resources and union representatives*		4	School 7 has conducted a work environment assessment among some staff: *In spring 2022, the principal carried out the survey jointly with human resources”*. Results are presented to relevant staff: *when the assessment was complete and analyzed.* [By] *school management orally in the work teams*. The school follows up on the action plan: *in the work teams*	5

n = number of schools within the specified category. Some schools did not achieve fidelity within certain recommendations, which explains why there is a discrepancy between the number of schools that achieved fidelity and the number of schools that were randomized into the two groups

For recommendation 3, the recipient perspective showed improvements in item 3A (absolute change multifaceted group = 8.9%, discrete group = 6.0%), meaning that a work environment survey had been conducted. Improvements in items 3B to 3I were observed in favor of the multifaceted schools (Table 4). From the implementers’ perspective, 35% (*n* = 10) of schools in the multifaceted group achieved high fidelity, compared with 7% (*n* = 2) in the discrete group (Table 6). Overall, 85% (*n* = 24) of schools in the multifaceted group reached fidelity at any level (high, moderate, modest, or low), whereas 61% (*n* = 16) of schools in the discrete group reached fidelity at any level (Table 6). The implementers’ perspective further showed staff involvement in the different activities among high-fidelity schools. For example, “*short-term goals were created with respective work teams.”* These schools also described concrete actions in an action plan and described in detail how they work with their action plans. For example: “*We still work with communication and that we are each other’s work environment. Some parts were improved in the Pulse survey* [a short work environment survey].*”* They also described when they follow up on the action plan (Table 6)*.* This illustrates how high-fidelity schools actively execute activities and follow up on progress, moving beyond merely creating a plan and establishing an ongoing process, as intended for working in accordance with recommendation 3. The results also showed that the multifaceted strategy was more effective in improving fidelity to this recommendation (Table 5), further supported by insights from the implementer’s perspective (Table 6).

## Discussion

This study aimed to compare the effectiveness of a multifaceted versus a discrete implementation strategy on fidelity to the *Guideline for the prevention of mental ill-health at the workplace*. In line with our hypothesis, the main finding is that the multifaceted strategy was more effective in improving fidelity than the discrete strategy. From the recipients’ perspective, the multifaceted strategy was more effective than the discrete strategy in seven out of ten fidelity indicators, each representing a specific target activity of the guideline. Moreover, the implementer perspective showed that more schools in the multifaceted group achieved high fidelity at 12 months across all three guideline recommendations compared to schools in the discrete group, as well as more schools in the multifaceted group achieved fidelity at any level when compared to the schools in the discrete group at 12 months. The findings further show that assessing fidelity from two perspectives provided complementarity and expansion of study findings in line with the hypothesis.

### The comparative effectiveness of the implementation strategies

The recipient perspective showed that the multifaceted strategy was more effective in improving fidelity than the discrete strategy. In the present trial, we were able to confirm our previous positive trends in fidelity [33] with statistically significant effects in favor of the multifaceted strategy. In line with these findings, the implementer perspective showed that more multifaceted schools achieved high fidelity compared to discrete schools. This suggests that adding facilitation to the multifaceted strategy increased fidelity to the guideline. In line with mechanistic research on facilitation [39, 40], facilitation is needed to provide implementation teams with the opportunity (resources and infrastructure) to implement the guideline, while the other components of the multifaceted strategy are hypothesized to provide teams with capability (knowledge and skills) and motivation [35], which in turn are hypothesized to improve fidelity. These hypothesized chain of events from strategy to fidelity are in accordance with the findings of a recent meta-analysis reporting associations between implementation strategies, COM-B-related determinants, and fidelity in school settings [66]. Future studies within the current trial will explore the different causal pathways through which the multifaceted implementation strategy affects fidelity.

Results from the implementer perspective showed that high-fidelity schools performed the target activities with more quality and rigor compared to low-fidelity schools, meaning that they had more staff involvement and continuous activities. However, a variation was observed between schools regarding the target activities that were enacted, resulting in three to four different fidelity levels per recommendation. This is in line with studies on organizational interventions showing variance in implementation across participating organizations [67, 68]. However, no variance was observed between schools belonging to the same fidelity level, irrespective of whether schools received the multifaceted or discrete implementation strategy. For some schools belonging to the discrete group, it was sufficient to have participated in the educational meeting to attain high fidelity. That the same level of fidelity can be attained independently of the strategy highlights the potential influence of school readiness to change, serving as a prerequisite for the school’s psychosocial risk management [69]. A less intense strategy, such as an educational meeting, might be sufficient for these schools to enact the relevant target activities of the recommendations. In line with this, Geng and colleagues [70] suggest that there is a need to explore whether strategies can be used sequentially. The sequential use of strategies allows for adaptations based on the response to the earlier strategy, so-called “on-the-go adaptations.” To meet the needs in resource-constrained educational settings, future studies should explore adaptive approaches that can make the implementation of the guideline more efficient [70–72].

Taken together, although the results acknowledge the potential avenue for adaptation of the multifaceted strategy, the findings of this study overall build a case for the need for a multifaceted strategy in this context. First and foremost, results from the recipient’s perspective showed statistically significant differences between the multifaceted and discrete groups, although the observed effect sizes were small overall. However, it is important to bear in mind that the relatively small changes reflect the extent to which school staff observed the changes implemented and can therefore be seen as a more distal outcome of implementation success. Second, the small effect sizes should be interpreted in light of the fact that the discrete group received two of the five components of the multifaceted strategy (educational meetings and implementation teams) and showed positive absolute changes in two of the three recommendations. The observed group differences may therefore be small, and effects could have been larger relative to a pure control group. The magnitude of the absolute changes observed within the multifaceted group does therefore provide an indicator of the meaningful improvements over time of the multifaceted strategy. Moreover, the observed relative changes suggest meaningful differences between the groups, underscoring the benefit of the multifaceted strategy over the discrete strategy. Thirdly, while the multifaceted strategy is more resource-intensive, its added practical value appears most evident for complex and ongoing change processes, such as in Recommendation 3, because it matches the level of complexity required by that recommendation. Our findings illustrated how, unlike discrete standalone strategies, this multifaceted strategy package worked synergistically to cumulatively provide the support that schools needed to achieve guideline fidelity. Leveraging the advantages of a qualitative approach, we also illustrated what high guideline fidelity looked like in practice from the perspectives of school management. The multifaceted schools improved their fidelity to all items of recommendation 3, whilst discrete schools decreased their fidelity to nearly all items of recommendation 3. This finding has practical relevance and addresses core challenges in schools’ management of psychosocial risks. A national evaluation of Swedish schools conducted by the Swedish Work Environment Authority showed that the main challenges schools faced with their systematic work environment were related to recommendation 3, specifically the lack of written action plans describing changes that need to be made in the work environment [73]. Similar findings were observed from the implementer perspective, 35.7% of the multifaceted schools attained high fidelity to recommendation 3 compared to only 7.4% of the discrete schools. Implementing recommendation 3, which involves multiple target activities that need to be enacted over time, likely needs more implementation support than provided by the discrete strategy. The improvements observed in favor of the multifaceted strategy for item 1C and recommendation 2 are most likely a result of the additional effect of the workshops, PDSA and facilitation and of schools actively engaging school staff and exposing them to the implemented changes as recommended in all three recommendations. For example, in those instances when school management has actively applied the knowledge that they have gained regarding the prevention of MHP within their school. Overall, our findings are in line with a recent systematic review showing that the effectiveness of organizational interventions to improve the psychosocial work environment, as recommended in the guideline, relies on complex and time-consuming implementation strategies, which should include both employees and their managers [37]. As shown in our previous trial, adhering to the guideline recommendations has the potential to impact school staffs’ work environment and health [34], underscoring the potential benefit of the observed improvements in the present trial.

### Examining and comparing fidelity from two perspectives

Assessing fidelity from two perspectives has helped us to draw valid conclusions about the effectiveness of the implementation strategies in fidelity attainment. The combination of perspectives provided a thorough understanding of fidelity as a multidimensional concept going beyond the implementers’ perspective of just delivering an intervention [44, 74, 75]. We observed agreement between the two perspectives – validating our findings regarding the comparative effectiveness of the multifaceted strategy and specifically for recommendation 3. The two perspectives also provided complementary information, expanding the findings of our trial. The recipient perspective provided a quantification of the change in fidelity over the 12-month follow-up period. By collecting information on fidelity from a large representative proportion of the school staff, a generalizable conclusion can be drawn that the changes made by the implementers were observed by the majority of staff. The implementer perspective provided detailed additional contextual information on the activities that were enacted in relation to these changes, offering a better understanding of the “how” in the implementation process. The implementer perspective highlighted the variability in how schools implemented changes into practice, suggesting that implementers reacted differently to the implementation strategies received. This underscores the importance of further assessing the enactment of activities to understand the process leading to the intended effect of the implementation strategies [44]. Moreover, it suggests that for some schools, adaptation of the strategies during the implementation process might be needed to enhance fidelity. This is in line with Chambers and colleagues’ [76] proposal that ongoing adaptations and refinements are necessary to enhance the fit of multi-level interventions to changing contexts and population needs. Future studies should explore how adaptations can be made in response to local needs or context, while preserving the strategies’ core elements or functions [71, 77].

### Implications for research and practice

This study addresses the research agenda for implementation outcomes research 2022–2023 [41] and issues related to the measurement and assessment of fidelity as an implementation outcome [44, 45]. It addresses the lack of studies that have used rigorous study designs, such as cluster-randomized trials, to test the comparative effectiveness of implementation strategies on implementation outcomes. The current study also provides knowledge on the effectiveness of implementation strategies in a less frequently studied setting, namely schools [78]. Longitudinal quantitative data is needed to gather evidence on the effectiveness of implementation strategies on fidelity and to test causal pathways to understand mechanisms of change. This type of data is more easily collected from recipients with a pre-post short survey, which allows for a more “objective” distal outcome, as it captures observed changes by those not directly involved in the execution of those changes. However, to gain a better understanding of how fidelity was attained, information is needed from those individuals who have been involved in the implementation process. The implementers can provide a detailed description of the “how” and “why” (behaviors) of the implementation process, as well as a description of how the enacted actions fit in their context. It allows for an investigation of not only the level of implementation, but also of the actions that have caused it to function. This is a valuable contribution to the operationalization of fidelity as a behavioral implementation outcome [79].

Furthermore, the fidelity checklist can fulfill purposes other than solely assessing fidelity as an outcome measure. First, the checklist can be used to continuously monitor fidelity during the implementation process, with a feedback loop back to the implementers to inform and improve implementation [80]. It can guide a sequential use of implementation strategies, enhancing efficiency and reducing the costs of the implementation [70, 71]. Using the checklist in longitudinal data collection may also provide insights into the time it takes for fidelity attainment, further addressing gaps identified in the implementation outcomes research agenda regarding the need to capture implementation outcome changes over time and the speed of their attainment [81].

Despite the important contribution of assessing fidelity from two perspectives, we also acknowledge related challenges. Applying the double perspective is a potentially more complex and time-consuming approach. Analyzing the open-ended questions from the checklist and categorizing them into different levels of fidelity has been challenging due to the diversity of reporting, with some descriptions of actions being very elaborate whilst others being very concise. An additional challenge concerns the timing of reporting. In the present study, the checklist completed at 12-month follow up was used to assess fidelity attainment. As there is a potential risk of recall bias [82], future studies could explore whether the checklist could be completed during the implementation process to better capture changes in fidelity. Moreover, there is a potential risk of recall bias from the recipient perspective. However, as organizational changes as recommended in the guideline often take time to implement, these changes are likely not observed by school staff within a shorten timeframe than 12-months was applied in the current trial [83, 84].

### Methodological considerations

A strong feature of this study is the cluster-randomized waiting-list controlled design that allows for experimental comparison of implementation strategies’ contribution to fidelity to the guideline [85, 86]. Another strength is the large sample of schools from four different municipalities representing a variety of sizes, organizations, sociodemographic and geographical aspects. The high response rate allows for generalizability to other schools in Sweden.

However, the study has limitations. One limitation is related to the time it takes to complete the checklist. Several reminders were sent to the schools to return the completed checklist. The schools were instructed to complete the checklist together with relevant staff, which likely made completion more time-consuming. Moreover, we believe that measuring fidelity from the perspective of staff provides valuable insights into the effectiveness of the implementation strategies, particularly since the measure is distal. This is especially important in this context, where studies suggest that meaningful changes in health require staff observation and acknowledgment of these changes [48, 53]. However, we must also acknowledge the potential limitation of this measure, as it relies on staff perceptions of organizational behavior. Staff may not be fully aware of all activities in hierarchical settings [87]. Another limitation is that the trial was conducted during the COVID-19 pandemic. In Sweden, compulsory schools were open during the pandemic and teaching continued as usual. There were, however, restrictions in place regarding large meetings. For some schools, this meant that not all staff could meet in person at the same time, for example during staff meetings. Other consequences of the pandemic were related to the workshops. Two of the workshops were conducted digitally. Some schools considered this a positive change, as participants could attend the workshops from their school, saving commuting time. Moreover, in one of the municipalities two workshops were given simultaneously in a shortened version, not compromising the content of the workshops.

## Conclusion

This study compared the effectiveness of two implementation strategies on fidelity to the *“guideline for the prevention of mental ill-health at the workplace”* in Swedish schools. The main conclusion is that the multifaceted implementation strategy was more effective than the discrete strategy in improving fidelity after 12 months. Assessing fidelity from the perspective of the implementers and recipients provided an understanding of the contextual functioning of the strategies, highlighting the variation in fidelity attainment and the importance to further examine the need for adaptations of strategies during the implementation process. Future studies within this trial will further explore the mechanisms underlying these strategies. This includes testing whether capability-, opportunity-, and motivation-related mediators account for the effect of the implementation strategies on fidelity, as well as leveraging implementation team members’ experiences to understand how individual strategies worked for the schools. For example, this might involve examining whether workshops facilitated skill acquisition and whether the PDSA strategy helped regulate the implementation process.

## Supplementary Information

Supplementary Material 1. CONSORT extension to cluster randomized trials.

## Data Availability

Not applicable.

## Abbreviations

PDSA: Plan-Do-Study-Act
MHP: Mental Health Problems
LMM: Linear Mixed Modeling
